# *H*_∞_ state estimation of continuous-time neural networks with uncertainties

**DOI:** 10.1038/s41598-024-52209-x

**Published:** 2024-01-22

**Authors:** Aiting Li, Yanhui Chen, Yun Hu, Dazhi Liu, Jinhui Liu

**Affiliations:** National Demonstration Center for Experimental Electronic Information and Telecommunication Engineering Education, Xidian University, Xi’an, 710071 China

**Keywords:** Electrical and electronic engineering, Communication and replication

## Abstract

$H_{\infty }$ state estimation is addressed for continuous-time neural networks in the paper. The norm-bounded uncertainties are considered in communication neural networks. For the considered neural networks with uncertainties, a reduced-order $H_{\infty }$ state estimator is designed, which makes that the error dynamics is exponentially stable and has weighted $H_{\infty }$ performance index by Lyapunov function method. Moreover, it is also given the devised method of the reduced-order $H_{\infty }$ state estimator. Then, considering that sampling the output *y*(*t*) of the neural network at every moment will result in waste of excess resources, the event-triggered sampling strategy is used to solve the oversampling problem. In addition, a devised method is also given for the event-triggered reduced-order $H_{\infty }$ state estimator. Finally, by the well-known Tunnel Diode Circuit example, it shows that a lower order state estimator can be designed under the premise of maintaining the same weighted $H_{\infty }$ performance index, and using the event-triggered sampling method can reduce the computational and time costs and save communication resources.

## Introduction

The state estimation or filtering issue^1–3^ has gained increasing attention in communication neural networks over the past few decades. In many applications, such as information processing and control engineering, it is common for large-scale neural networks to have only partial access to information from the network output. Thus, to estimate the neuron state must be estimated from the available output measurements of the network and then utilized to implement a specific scenario.^4–17^ The purpose for state estimation is to estimate the internal state values using the measured outputs of communication neural networks. Specifically, the outputs of neural networks are used as the inputs to devise a state estimator such that the error dynamic outputs are robust to external noise with reference to the output errors of the original neural networks. As a tool widely used to solve state estimation issues, while $H_{\infty }$ state estimation makes no additional statistical assumptions about exogenous input signals compared to orthodox state estimators^18^ likely the Kalman state estimator. Besides, uncertainty can cause instability in neural network systems, come down to epistemic situations concerning imperfect or unknown information^19,20^. And the reduced-order state estimation is a the useful method to implement state estimation and save computing resources^21^. Therefore, for the reduced-order $H_{\infty }$ state estimation of neural networks applied in the field of communication, it is vital to consider with uncertainty.

Stability^22–24^, which describes whether the plant has convergence under initial conditions (not necessarily zero), is independent of the input action. And stability is the basis for the work of neural networks. The stability theory proposed by Lyapunov in 1892 is highly superior in the study of stability^25^: for internal descriptive models; for univariate, linear, constant; for multivariate, nonlinear, time-varying systems. Lyapunov functional method is a simple and useful method to study stability of neural networks with uncertainties^26–29^.

When the neural network is applied to information transmission, it is necessary to sample the data from time to time, although it is possible to transmit all the data resources, sometimes unnecessary data is also transmitted, which can waste the communication resources.But event-triggered control can increase efficiency while guaranteeing performance^30,31^. Most of the studies on controlling the periodic execution of events for signals in communication transmission systems has been through ZOH or time-event-triggered schemes^32,33^. From the theoretical analysis point of view, the time-triggered scheme and ZOH are preferred. The time-triggered sampling strategy proposed in this paper^34–36^ is a good solution to the problem of wasted communication resources^37^. It samples only under the condition of “event” occurrence, so it can save resources by designing appropriate “event triggering conditions” for sampling and avoiding unnecessary data transmission. Therefore, it is necessary to design an event-triggered reduced-order $H_{\infty }$ state estimator.

In this paper, it is solved $H_{\infty }$ state estimation of continuous-time neural networks with uncertainties. This is the first time that we greatly popularized the reduced-order and event-triggered reduced-order $H_{\infty }$ state estimator to continuous-time neural networks with uncertainties and considered about its information transmission performance. The main contributions are as follows: (1) For the continuous-time neural networks with bounded uncertainties, a reduced $H_{\infty }$ state estimator is addressed using the outputs of the considered neural networks for the goal of state estimation. (2) Some sufficient conditions in the form of LMIs are provided to make sure that the error system is exponentially stable and has weighted $H_{\infty }$ performance index by Lyapunov function method. (3) In addition, an event-triggered strategy is structured for the outputs’ sampling of the considered neural networks in order to reduce the number of outputs’ samples. (4) Based on the structured event-triggered strategy, a reduced $H_{\infty }$ state estimator is also addressed using the event-triggered outputs of the considered neural networks for the goal of state estimation. (5) And there is given some sufficient conditions in LMIs form which can guarantee the exponentially stability with the same weighted $H_{\infty }$ performance index for the error system. (6) Finally, compared the usual reduced $H_{\infty }$ state estimator with the reduced $H_{\infty }$ state estimator based on the structured event-triggered strategy in application of the well-known Tunnel Diode Circuit example, the latter has fewer sampling times than the former, thus achieving the purpose of saving computer resources.

This study is formed as below. In “Problem statements and preliminaries”, it is provided some preliminary results. The $H_{\infty }$ state estimation issue is discussed in “Main results” for continuous-time neural networks with uncertainties. Some simulations are elucidated in “Simulation” the reasonability of the presented methods. And “Conclusions” is concluded this study.

Notations: $\mathbb {R}^n$ is the *n* dimensional vectors space, $\mathbb {R}^{n\times m}$ is all $(n\times m)$ dimensional real matrices set. With regard to $P\in \mathbb {R}^{n\times n}$, $P>0$, $P^{-1}$ and $P^T$ are represented respectively that *P* is symmetric positive definite matrix, the inverse and transpose of *P*. The symmetrical items is denoted by $*$ in a symmetric matrix. For $P\in \mathbb {R}^{n\times n}$, $\lambda _{min}(P)$ is the minimum eigenvalue and $\lambda _{max}(P)$ is maximum eigenvalue. *I* and 0 are the identity and zero matrix with suitable dimensions, respectively. $\mathbb {L}_{2}[0,\infty )$ expresses the square integrable function space over $[0,\infty )$. $\Vert ~\Vert$ means the norm of Euclidean.

## Problem statements and preliminaries

Consider continuous-time neural networks with uncertainties as following:

1 $$\begin{aligned} \begin{aligned} \dot{e}(t)&=(A+\Delta A(t))e(t)+(B+\Delta B(t))f(e(t))+Cv(t), \\ y(t)&=De(t)+Ff(e(t))+Gv(t), \\ z(t)&=He(t), \\ \end{aligned} \end{aligned}$$

where $e(t)\in \mathbb {R}^{n}$ is the system state, $$f(e(t))=\begin{bmatrix} f_{1}(e_{1}(t))&f_{2}(e_{2}(t))&\cdots&f_{n}(e_{n}(t)) \end{bmatrix}^{T}\in \mathbb {R}^{n}$$ is the neuron activation function, $y(t)\in \mathbb {R}^{m}$ is the measured output, $z(t) \in \mathbb {R}^{p}$ is the estimated signal, $v(t)\in \mathbb {R}^{q}$ denotes the Gaussian white noise, $v(t)\in \mathcal {L}_{2}[0,\infty )$. $A\in \mathbb {R}^{n\times n}$, $B\in \mathbb {R}^{n\times n}$, $C\in \mathbb {R}^{n\times q}$, $D\in \mathbb {R}^{m\times n}$, $F\in \mathbb {R}^{m\times n}$, and $G\in \mathbb {R}^{m\times q}$ are the known constant matrices. $\Delta A(t)$, $\Delta B(t)$ represent the system time-varying uncertainties which are unknown matrices and subject to the below constraints

2 $$\begin{aligned} \begin{bmatrix}\Delta A(t)&\Delta B(t)\end{bmatrix} =MN(t)\begin{bmatrix}L_{1}&L_{2}\end{bmatrix} \end{aligned}$$

where $M\in \mathbb {R}^{n\times n}$, $L_{1},~L_{2}\in \mathbb {R}^{n\times n}$ are matrices of set constants, and *N*(*t*) satisfying $N^{T}(t)N(t)\le I$ is an unknown time-varying matrix. For ease of notation, set $\Delta A(t)\triangleq \Delta A$ and $\Delta B(t)\triangleq \Delta B$.

**Figure 1:**
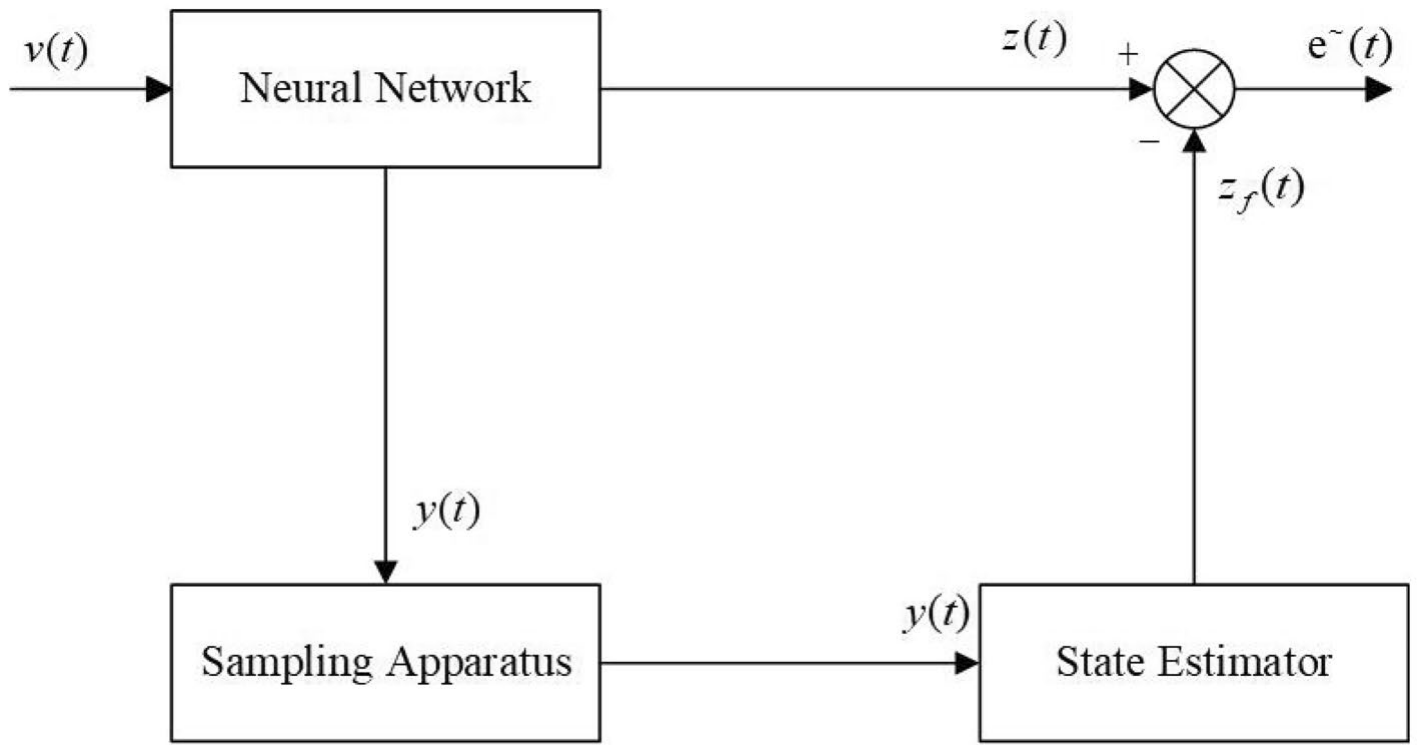
The Structure for State Estimator of the system (1).

Then for the system (1), the $H_{\infty }$ state estimator is formed as

3 $$\begin{aligned} \begin{aligned} \dot{e}_{f}(t)&=A_{f}e_{f}(t)+B_{f}y(t), \\ z_{f}(t)&=C_{f}e_{f}(t)+D_{f}y(t), \\ \end{aligned} \end{aligned}$$

in which $e_{f}(t)\in \mathbb {R}^{n_{f}}$ is the state of estimator and $z_{f}(t)\in \mathbb {R}^{p}$ is estimated *z*(*t*), $A_{f}\in \mathbb {R}^{n_{f}\times n_{f}}$, $B_{f}\in \mathbb {R}^{n_{f}\times m}$, $C_{f}\in \mathbb {R}^{p\times n_{f}}$, and $D_{f}\in \mathbb {R}^{p\times m}$ are the unknown filter matrices. And $1\le n_{f}\le n$, when $n_{f}=n$, Eq. (3) is called the full-order $H_{\infty }$ state estimator of Eq. (1); when $1\le n_{f}< n$, Eq. (3) is called the reduced-order $H_{\infty }$ state estimator of Eq. (1). And the structure of the plant is shown in Fig. 1.

From Eqs. (1) and (3), it is gotten the error dynamics

4 $$\begin{aligned} \begin{aligned} \dot{\xi }(t)&=(\tilde{A}+\Delta \tilde{A})\xi (t) +(\tilde{B}+\Delta \tilde{B})f(e(t))+\tilde{C}v(t), \\ \tilde{e}(t)&=\tilde{H}\xi (t)-D_{f}Ff(e(t)) -D_{f}Gv(t), \\ \end{aligned} \end{aligned}$$

where $$\xi (t)=\left[ \begin{array}{l} e(t) \\ e_{f}(t) \\ \end{array} \right] $$,  $$\tilde{A}=\left[ \begin{array}{ll} A &{} 0 \\ B_{f}D &{} A_{f}\\ \end{array} \right] $$,  $$\Delta \tilde{A}=\left[ \begin{array}{ll} \Delta A &{} 0 \\ 0 &{} 0\\ \end{array} \right] $$,  $$\tilde{B}=\left[ \begin{array}{ll} B \\ B_{f}F \\ \end{array} \right] $$,  $$\Delta \tilde{B}=\left[ \begin{array}{ll} \Delta B \\ 0 \\ \end{array} \right] $$,  $$\tilde{C}=\left[ \begin{array}{ll} C \\ B_{f}G\\ \end{array} \right] $$, 

$$\tilde{H}=\left[ \begin{array}{ll} H-D_{f}D &{} -C_{f}\\ \end{array} \right] $$,  $$\tilde{e}(t)=z(t)-z_{f}(t)$$.

And the lemmas and definition are also proposed as following.

### Lemma 1

(^32^ Schur complement lemma) *For a given symmetric matric* $$S=\left[ \begin{array}{ll} S_{11} &{} S_{12} \\ S_{12}^{T} &{} S_{22} \\ \end{array} \right] \in \mathbb {R}^{(n+m)\times (n+m)}$$, *where* $S_{11}\in \mathbb {R}^{n\times n}$ *and* $S_{22}\in \mathbb {R}^{m\times m}$, *then the following three statements are equivalent*:

- $$S<0$$;
- $$S_{11}<0$$, $$S_{22}-S_{12}^{T}S_{11}^{-1}S_{12}<0$$;
- $$S_{22}<0$$, $$S_{11}-S_{12}S_{22}^{-1}S_{12}^{T}<0$$.

### Lemma 2

^17^ *For matrices* *M*, *L* *and* *N*(*t*) *with proper dimensions*, *N*(*t*) *satisfies* $N^{T}(t)N(t)\le I$, *then it holds*

$$\begin{aligned} M^{T}N(t)L+L^{T}N^{T}(t)M\le \iota M^{T}M+\iota ^{-1}L^{T}L,~ 0<\iota \in \mathbb {R}. \end{aligned}$$

### Lemma 3

^23^ *Dispute Eq*. (4) *with* $v(t)\equiv 0$. *Let* $\xi (0)=0$ *be the equilibrium point of it, assume that there exists a Lyapunov functional* $V(t, \xi (t))$ *and class*-$\kappa$ *functions* $\kappa _{i}$, $i=1, 2, 3$ *satisfying*

$$\begin{aligned} \kappa _{1}(\Vert \xi (t)\Vert )\le \dot{V}(t, \xi (t))\le \kappa _{2}(\Vert \xi (t)\Vert ), \end{aligned}$$

*and*

$$\begin{aligned} \dot{V}(t, \xi (t))\le -\kappa _{3}(\Vert \xi (t)\Vert ), \end{aligned}$$

*then the system* (4) *is asymptotically stable. If* $\kappa _{3}(\Vert \xi (t)\Vert )$ i*s decreasing exponentially, then the system* (4) *is exponentially stable*.

### Definition 1

For a preassigned constant $\gamma >0$, the system (4) is called to be exponentially stable and have a weighted $H_{\infty }$ performance index $\gamma$, if it satiafies

1. when $v(t)=0$, the system (4) is exponentially stable;
2. under zero initial condition (ZIC), one holds
  $$\begin{aligned} \int _{0}^{\infty }\tilde{e}^{T}(t)\tilde{e}(t)dt \le \gamma ^{2}\int _{0}^{\infty }v^{T}(t)v(t)dt \end{aligned}$$

when $$v(t)\in \mathcal {L}_{2}[0, \infty )$$.

## Main results

The $H_{\infty }$ state estimation issue here is to devise the state estimator matrices $A_{f}$, $B_{f}$, $C_{f}$, and $D_{f}$ to make sure the system (4) be exponentially stable and have a weighted $H_{\infty }$ performance index. The main results are as following.

### Theorem 1

*For the system* (4) *and the given positive constant* $\gamma _{0}$, *suppose there exist positive scalars* $\iota _{1}$, $\iota _{2}$, *and a positive symmetric matrices* $P_{1i}\in \mathbb {R}^{n\times n}$, $P_{2j}\in \mathbb {R}^{n_{f}\times n_{f}}$, *matrices* $A_{F}\in \mathbb {R}^{n_{f}\times n_{f}}$, $B_{F}\in \mathbb {R}^{n_{f}\times m}$, $C_{F}\in \mathbb {R}^{p\times n_{f}}$, and $D_{F}\in \mathbb {R}^{p\times m}$ *such that*

5 $$\begin{aligned} \left[ \begin{array}{cccccccccc} P_{1}A+A^{T}P_{1}+\iota _{1}L_{1}^{T}L_{1}+2P_{1} &{} D^{T}B_{F}^{T} &{} P_{1}B &{} P_{1}C &{} H^{T}-D^{T}D_{F}^{T} &{} P_{1}M \\ *&{} A_{F}+2P_{2} &{} B_{F}F &{} B_{F}G &{} -C_{F}^{T} &{} 0\\ *&{} *&{} -\iota _{2}L_{2}^{T}L_{2} &{} 0 &{} -F^{T}D_{F}^{T} &{} 0\\ *&{} *&{} *&{} -\gamma _{0}^{2}I &{} -G^{T}D_{F}^{T} &{} 0\\ *&{} *&{} *&{} *&{} -I &{} 0\\ *&{} *&{} *&{} *&{} *&{} -\frac{1}{\iota _{1}^{-1}+\iota _{2}^{-1}}I\\ \end{array} \right] <0 \end{aligned}$$

*hold, then the system* (4) *be exponentially stable and have a weighted* $H_{\infty }$ *performance index* $\gamma _{0}$. *And the state estimator matrices are* $A_{f}=P_{2}^{-1}A_{F}$, $B_{f}=P_{2}^{-1}B_{F}$, $C_{f}=C_{F}$, *and* $D_{f}=D_{F}$.

### Proof

Let the Lyapunov function be

6 $$\begin{aligned} V(t, \xi (t))\triangleq V(t)=\xi ^{T}(t)P\xi (t),~ P=\begin{bmatrix}P_{1} &{} 0 \\ 0 &{} P_{2} \end{bmatrix}, \end{aligned}$$

then it obtains

7 $$\begin{aligned} a\Vert \xi (t)\Vert ^{2}\le V(t)\le b\Vert \xi (t)\Vert ^{2},~ \forall t\ge 0, \end{aligned}$$

in which $$a=\min \{\lambda _{min}(P_{1}), \lambda _{min}(P_{2})\}$$, $$b=\max \{\lambda _{max}(P_{1}), \lambda _{max}(P_{2})\}$$.

Then, it will be proved that

8 $$\begin{aligned} \begin{aligned} \dot{V}(t)+2V(t)+\tilde{e}^{T}(t)\tilde{e}(t)-\gamma ^{2}_{0}v^{T}(t)v(t)\le 0, ~ \forall t\ge 0. \end{aligned} \end{aligned}$$

According to Eqs. (3) and (4), taking the time derivation of Lyapunov function (4) along (3) could be obtained that

9 $$\begin{aligned} \begin{aligned}{}&\dot{V}(t)+2V(t)+\tilde{e}^{T}(t)\tilde{e}(t)-\gamma ^{2}_{0}v^{T}(t)v(t) \\&\quad =\dot{\xi }^{T}(t)P\xi (t)+\xi ^{T}(t)P\dot{\xi }(t)+2\xi ^{T}(t)P\xi (t) +\tilde{e}^{T}(t)\tilde{e}(t)-\gamma ^{2}_{0}v^{T}(t)v(t) \\&\quad \le [(\tilde{A}+\Delta \tilde{A})\xi (t)+(\tilde{B}+\Delta \tilde{B})f(e(t)) +\tilde{C}V(t)]^{T}P\xi (t) +\xi ^{T}(t)P[(\tilde{A}+\Delta \tilde{A})\xi (t)+(\tilde{B}+\Delta \tilde{B})f(e(t)) +\tilde{C}V(t)] \\&\quad \quad +2\xi ^{T}(t)P\xi (t) +[\tilde{H}\xi (t)-D_{f}Ff(e(t))-D_{f}G(t)]^{T}[\tilde{H}\xi (t)-D_{f}Ff(e(t))-D_{f}G(t)] -\gamma ^{2}_{0}v^{T}(t)v(t) \\&\quad = \begin{bmatrix}e(t) \\ e_{f}(t)\end{bmatrix}^{T} \begin{bmatrix}A^{T}P_{1}+\Delta A^{T}P_{1} &{} D^{T}B_{f}^{T}P_{2} \\ 0 &{} A_{f}^{T}P_{2} \end{bmatrix} \begin{bmatrix}e(t) \\ e_{f}(t)\end{bmatrix} +f^{T}(e(t))\begin{bmatrix}B^{T}P_{1}+\Delta B^{T}P_{1}&F^{T}B_{f}^{T}P_{2}\end{bmatrix} \begin{bmatrix}e(t) \\ e_{f}(t)\end{bmatrix} \\&\quad \quad +v^{T}(t)\begin{bmatrix}C^{T}P_{1}&G^{T}B_{f}^{T}P_{2}\end{bmatrix} \begin{bmatrix}e(t) \\ e_{f}(t)\end{bmatrix} +\begin{bmatrix}e(t) \\ e_{f}(t)\end{bmatrix}^{T} \begin{bmatrix}P_{1}A+P_{1}\Delta A &{} 0 \\ P_{2}B_{f}D &{} P_{2}A_{f} \end{bmatrix} \begin{bmatrix}e(t) \\ e_{f}(t)\end{bmatrix} \\&\quad \quad +\begin{bmatrix}e(t) \\ e_{f}(t)\end{bmatrix}^{T} \begin{bmatrix}P_{1}B+P_{1}\Delta B \\ P_{2}B_{f}F\end{bmatrix} f(e(t)) +\begin{bmatrix}e(t) \\ e_{f}(t)\end{bmatrix}^{T} \begin{bmatrix}P_{1}C \\ P_{2}B_{f}G\end{bmatrix}v(t) +2\begin{bmatrix}e(t) \\ e_{f}(t)\end{bmatrix}^{T} \begin{bmatrix}P_{1} &{} 0 \\ 0 &{} P_{2}\end{bmatrix}^{T} \begin{bmatrix}e(t) \\ e_{f}(t)\end{bmatrix} \\&\quad \quad +[\tilde{H}\xi (t)-D_{f}Ff(e(t))-D_{f}G(t)]^{T}[\tilde{H}\xi (t)-D_{f}Ff(e(t))-D_{f}G(t)] -\gamma ^{2}_{0}v^{T}(t)v(t) \\&\quad \le \eta ^{T}(t)(\Omega _{1}+\Omega _{2}^{T}\Omega _{2})\eta (t) \end{aligned} \end{aligned}$$

where $$\eta (t)=\begin{bmatrix} e(t) \\ e_{f}(t) \\ f(e(t)) \\ v(t) \\ \end{bmatrix}$$,  $$\Omega _{1}=\begin{bmatrix} P_{1}A+A^{T}P_{1}+2P_{1}+\iota _{1}L_{1}^{T}L_{1} +(\iota _{1}^{-1}+\iota _{2}^{-1})P_{1}MM^{T}P_{1} &{} D^{T}B_{F}^{T} &{} P_{1}B &{} P_{1}C \\ *&{} A_{F}+2P_{2} &{} B_{F}F &{} B_{F}G \\ *&{} *&{} -\iota _{2}L_{2}^{T}L_{2} &{} 0 \\ *&{} *&{} *&{} -\gamma _{0}^{2}I \\ \end{bmatrix}$$, 

$$\Omega _{2}=\begin{bmatrix} H-D_{F}D&-C_{F}&-D_{F}F&-D_{F}G \end{bmatrix}$$,  $$A_{F}=P_{2}A_{f}$$,  $$B_{F}=P_{2}B_{f}$$,  $$C_{F}=C_{f}$$,  $$D_{F}=D_{f}$$.

The last step in Eq. (9) utilizes Lemma 2:

$$\begin{aligned} e^{T}(t)\Delta A^{T}P_{1}e(t)+e^{T}(t)P_{1}\Delta Ae(t) = & e^{T}(t)L_{1}^{T}N^{T}(t)M^{T}P_{1}e(t)+e^{T}(t)P_{1}MN(t)L_{1}e(t)\\ \le & \iota _{1}^{-1}e^{T}(t)P_{1}MM^{T}P_{1}e(t)+\iota _{1}e^{T}(t)L_{1}^{T}L_{1}e(t); \\ f^{T}(e(t))\Delta B^{T}P_{1}e(t)+e^{T}(t)P_{1}\Delta Bf(e(t)) =&f^{T}(e(t))L_{2}^{T}N^{T}(t)M^{T}P_{1}e(t)+e^{T}(t)P_{1}MN(t)L_{2}f(e(t))\\ \le & \iota _{2}^{-1}e^{T}(t)P_{1}MM^{T}P_{1}e(t)+\iota _{2}f^{T}(e(t))L_{1}^{T}L_{1}f(e(t)).\\ \end{aligned} $$

Then, using the Schur Complement Lemma twice for Eq. (6) and collapsing yields $\Omega _{1}+\Omega _{2}^{T}\Omega _{2}<0$, and thus Eq. (8) holds.

Applying Eq. (8) yields that

10 $$\begin{aligned} \begin{aligned} V(t,\xi (t)) \le \exp (-2t)V(0,\xi (0)) -\int _{0}^{t}\exp (-2(t-s))[\tilde{e}^{T}(s)\tilde{e}(s)-\gamma ^{2}_{0}v^{T}(s)v(s)]ds. \\ \end{aligned} \end{aligned}$$

When $v(t)=0$, utilizing Eq. (10) and $\tilde{e}^{T}(s)\tilde{e}(s)>0$, it is implied that

$$\begin{aligned} V(t,\xi (t)) \le \exp (-2t)V(0,\xi (0)). \end{aligned}$$

Combining it and Eq. (7), the system (4) is exponentially stable with $v(t)=0$ on the grounds of Lemma 3.

Now, consider $v(t)\ne 0$. Under ZIC, one has $V(0,\xi (0))=0$, $V(t)\ge 0$. Following Eq. (10), sequentially, it has

11 $$\begin{aligned} \begin{aligned} \int _{0}^{t}\exp (-2(t-s)-2s)\tilde{e}^{T}(s)\tilde{e}(s)ds \le \int _{0}^{t}\exp (-2(t-s))\tilde{e}^{T}(s)\tilde{e}(s)ds \le \int _{0}^{t}\exp (-2(t-s))\gamma ^{2}_{0}v^{T}(s)v(s)ds. \end{aligned} \end{aligned}$$

Furthermore, integrate *t* from 0 to $\infty$ on two sides of Eq. (11), it gets that

$$\begin{aligned} \int _{0}^{\infty }\tilde{e}^{T}(t)\tilde{e}(t)dt \le \gamma ^{2}_{0}\int _{0}^{\infty }v^{T}(t)v(t)dt. \end{aligned}$$

Thus, based on Definition 1, the system (4) is exponentially stable and has a weighted $H_{\infty }$ performance index $\gamma _{0}$. Proof is over. $\square$

**Figure 2:**
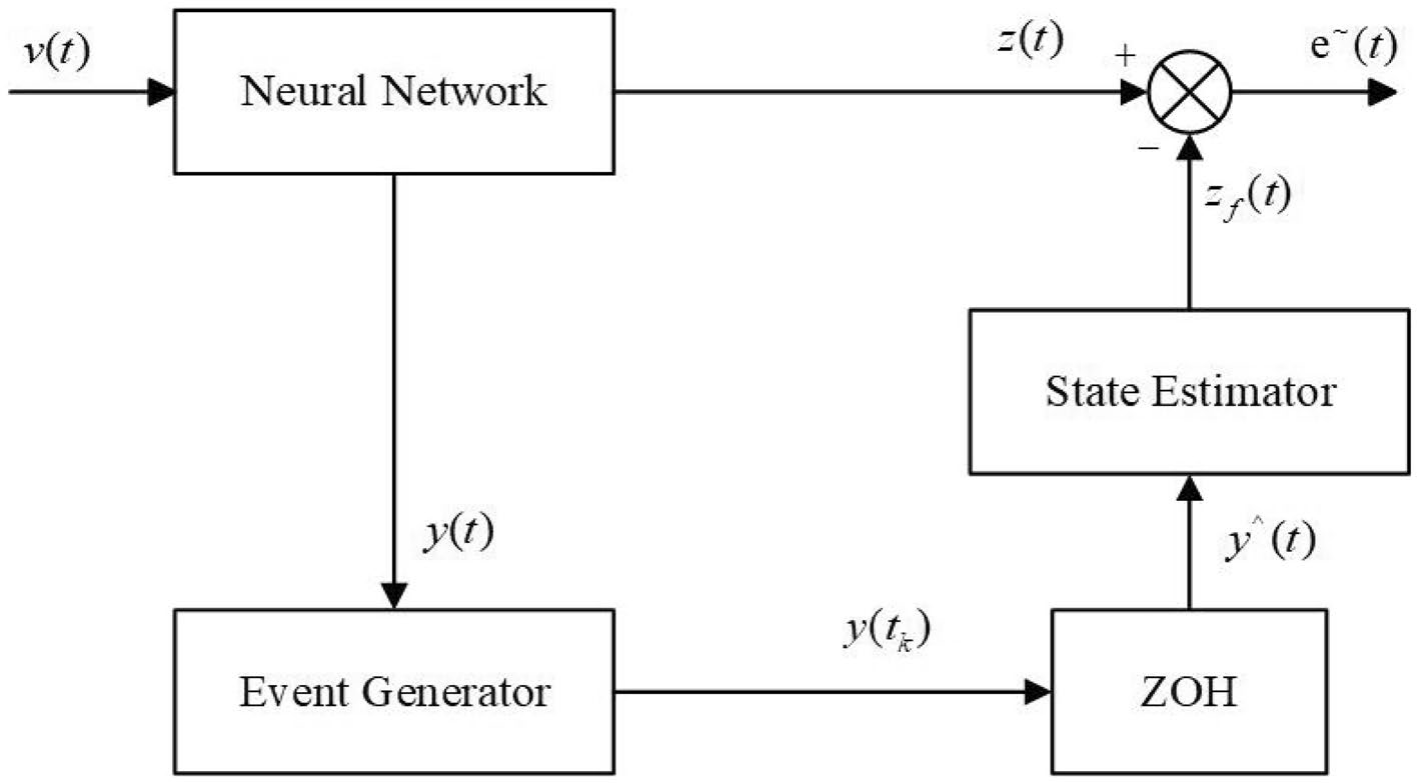
The structure for state estimator based on event-triggered sampling strategy (Eq. 12).

On the other hand, consider that sampling y at all times exists to sample unnecessary data. To determine the specific values of the outputs *y*(*t*) and reduce the number of samples, an event-triggered sampling strategy is used to generate the sampling time series $\{t_{k}\}$, $t_{0}=0$:

12 $$\begin{aligned} \begin{aligned} t_{k+1}=\{t>t_{k}\mid e_{y}^{T}(t)\Phi e_{y}(t)\ge y^{T}(t_{k})\Psi y(t_{k})\}, \\ \end{aligned} \end{aligned}$$

where $$e_{y}(t)=y(t)-y(t_{k})$$, $$t>t_{k}$$, $$\Phi >0$$ and $$\Psi >0$$ are event-triggered parameters to be designed. And in the event-triggered sampling interval $$[t_{k}, t_{k+1})$$, set $$\hat{y}(t)=y(t_{k})$$, where $$\hat{y}(t)$$ is the output of Zero Order Holder (ZOH) in Fig. 2. Then for the system (1), the form of the $$H_{\infty }$$ state estimator based on the event-triggered sampling strategy (12) is

13 $$\begin{aligned} \begin{aligned} \dot{e}_{f}(t)&=A_{f}e_{f}(t)+B_{f}\hat{y}(t), \\ z_{f}(t)&=C_{f}e_{f}(t)+D_{f}\hat{y}(t), \\ \end{aligned} \end{aligned}$$

where $e_{f}(t)$, $z_{f}(t)$, $A_{f}$, $B_{f}$, $C_{f}$, and $D_{f}$ are same as them in (3).

From Eqs. (1), (12) and (13), it is gotten the error dynamics

14 $$\begin{aligned} \begin{aligned} \dot{\xi }(t)&=(\tilde{A}+\Delta \tilde{A})\xi (t) +(\tilde{B}+\Delta \tilde{B})f(e(t))+\hat{B}e_{y}(t)+\tilde{C}v(t),\\ \tilde{e}(t)&=\tilde{H}\xi (t)-D_{f}Ff(e(t))+D_{f}e_{y}(t) -D_{f}Gv(t), \\ \end{aligned} \end{aligned}$$

where $\xi (t)$,  $\tilde{A}$,  $\Delta \tilde{A}$,  $\tilde{B}$,  $\Delta \tilde{B}$,  $\tilde{C}$,  $\tilde{H}$,  and $\tilde{e}(t)$ are same as them in (4), and $$\hat{B}=\left[ \begin{array}{ll} 0 \\ -B_{f} \\ \end{array} \right] $$.

Then, based on the event-triggered sampling strategy (Eq. 12), the solution of $H_{\infty }$ state estimation issue is as following:

### Theorem 2

*For the system* (14) *and the given positive constant* $\gamma _{0}$, *suppose there exist positive scalars* $\iota _{1}$, $\iota _{2}$, *and a positive symmetric matrices* $P_{1i}\in \mathbb {R}^{n\times n}$, $P_{2j}\in \mathbb {R}^{n_{f}\times n_{f}}$, $\Phi \in \mathbb {R}^{m\times m}$, $\Psi \in \mathbb {R}^{m\times m}$ *matrices* $A_{F}\in \mathbb {R}^{n_{f}\times n_{f}}$, $B_{F}\in \mathbb {R}^{n_{f}\times m}$, $C_{F}\in \mathbb {R}^{p\times n_{f}}$, *and* $D_{F}\in \mathbb {R}^{p\times m}$ *such that*

15 $$\begin{aligned} \left[ \begin{array}{cccccccccc} P_{1}A+A^{T}P_{1}+\iota _{1}L_{1}^{T}L_{1}+2P_{1} &{} D^{T}B_{F}^{T} &{} P_{1}B &{} 0 &{} P_{1}C &{} H^{T}-D^{T}D_{F}^{T} &{} D^{T}\Psi &{} P_{1}M \\ *&{} A_{F}+2P_{2} &{} B_{F}F &{} -B_{F} &{} B_{F}G &{} -C_{F}^{T} &{} 0 &{} 0\\ *&{} *&{} -\iota _{2}L_{2}^{T}L_{2} &{} 0 &{} 0 &{} -F^{T}D_{F}^{T} &{} F^{T}\Psi &{} 0\\ *&{} *&{} *&{} -\Phi &{} 0 &{} 0 &{} -\Psi &{} 0 \\ *&{} *&{} *&{} *&{} -\gamma _{0}^{2}I &{} -G^{T}D_{F}^{T} &{} G^{T}\Psi &{} 0\\ *&{} *&{} *&{} *&{} *&{} -I &{} 0 &{} 0\\ *&{} *&{} *&{} *&{} *&{} *&{} -\Psi &{} 0 \\ *&{} *&{} *&{} *&{} *&{} *&{} *&{} -\frac{1}{\iota _{1}^{-1}+\iota _{2}^{-1}}I\\ \end{array} \right] <0 \end{aligned}$$

*hold, then the system* (14) *be exponentially stable and have a weighted* $H_{\infty }$ *performance index* $\gamma _{0}$. *And the state estimator matrices are* $A_{f}=P_{2}^{-1}A_{F}$, $B_{f}=P_{2}^{-1}B_{F}$, $C_{f}=C_{F}$, *and* $D_{f}=D_{F}$.

### Proof

Also select the Lyapunov function as Eq. (6). Only the proof of the following equation is given here, and the rest of the proof procedure is similar to the proof of Theorem 1.

16 $$\begin{aligned} \begin{aligned} \dot{V}(t)+2V(t)+\tilde{e}^{T}(t)\tilde{e}(t)-\gamma ^{2}_{0}v^{T}(t)v(t)\le 0, ~ \forall t\in [t_{k}, t_{k+1}). \end{aligned} \end{aligned}$$

By the event-triggered sampling strategy (Eq. 12), when $t\in [t_{k}, t_{k+1})$, the event is not triggered, it means that

$$\begin{aligned} e_{y}^{T}(t)\Phi e_{y}(t)< y^{T}(t_{k})\Psi y(t_{k}),~t\in [t_{k}, t_{k+1}), \end{aligned}$$

i.e.

17 $$\begin{aligned} \begin{aligned} e_{y}^{T}(t)\Phi e_{y}(t)< (y(t)-e_{y}(t))^{T}\Psi (y(t)-e_{y}(t)),~t\in [t_{k}, t_{k+1}). \\ \end{aligned} \end{aligned}$$

In $$[t_{k}, t_{k+1})$$, according to Eqs. (13), (14) and (17), and taking the time derivation of Lyapunov function (6) along (14) could be obtained that

18 $$\begin{aligned}  & \dot{V}(t)+2V(t)+\tilde{e}^{T}(t)\tilde{e}(t)-\gamma ^{2}_{0}v^{T}(t)v(t) \\&\quad = \dot{\xi }^{T}(t)P\xi (t)+\xi ^{T}(t)P\dot{\xi }(t)+2\xi ^{T}(t)P\xi (t) +\tilde{e}^{T}(t)\tilde{e}(t)-\gamma ^{2}_{0}v^{T}(t)v(t) \\&\quad \le [(\tilde{A}+\Delta \tilde{A})\xi (t)+(\tilde{B}+\Delta \tilde{B})f(e(t)) +\hat{B}e_{y}(t)+\tilde{C}V(t)]^{T}P\xi (t) \\&\quad \quad +\xi ^{T}(t)P[(\tilde{A}+\Delta \tilde{A})\xi (t)+(\tilde{B}+\Delta \tilde{B})f(e(t)) +\hat{B}e_{y}(t)+\tilde{C}V(t)]+2\xi ^{T}(t)P\xi (t) \\&\quad \quad +[\tilde{H}\xi (t)-D_{f}Ff(e(t))+D_{f}e_{y}(t)-D_{f}G(t)]^{T} [\tilde{H}\xi (t)-D_{f}Ff(e(t))+D_{f}e_{y}(t)-D_{f}G(t)] \\&\quad \quad -\gamma ^{2}_{0}v^{T}(t)v(t) +(y(t)-e_{y}(t))^{T}\Psi (y(t)-e_{y}(t))-e_{y}^{T}(t)\Phi e_{y}(t)\\&\quad = \begin{bmatrix}e(t) \\ e_{f}(t)\end{bmatrix}^{T} \begin{bmatrix}A^{T}P_{1}+\Delta A^{T}P_{1} &{} D^{T}B_{f}^{T}P_{2} \\ 0 &{} A_{f}^{T}P_{2} \end{bmatrix} \begin{bmatrix}e(t) \\ e_{f}(t)\end{bmatrix} +f^{T}(e(t))\begin{bmatrix}B^{T}P_{1}+\Delta B^{T}P_{1}&F^{T}B_{f}^{T}P_{2}\end{bmatrix} \begin{bmatrix}e(t) \\ e_{f}(t)\end{bmatrix} \\&\quad \quad +e_{y}^{T}(t)\begin{bmatrix} 0&-B_{f}^{T}P_{2}\end{bmatrix} \begin{bmatrix}e(t) \\ e_{f}(t)\end{bmatrix} +v^{T}(t)\begin{bmatrix}C^{T}P_{1}&G^{T}B_{f}^{T}P_{2}\end{bmatrix} \begin{bmatrix}e(t) \\ e_{f}(t)\end{bmatrix}\\&\quad \quad +\begin{bmatrix}e(t) \\ e_{f}(t)\end{bmatrix}^{T} \begin{bmatrix}P_{1}A+P_{1}\Delta A &{} 0 \\ P_{2}B_{f}D &{} P_{2}A_{f} \end{bmatrix} \begin{bmatrix}e(t) \\ e_{f}(t)\end{bmatrix} +\begin{bmatrix}e(t) \\ e_{f}(t)\end{bmatrix}^{T} \begin{bmatrix}P_{1}B+P_{1}\Delta B \\ P_{2}B_{f}F\end{bmatrix} f(e(t)) \\&\quad \quad +\begin{bmatrix}e(t) \\ e_{f}(t)\end{bmatrix}^{T} \begin{bmatrix} 0 \\ -P_{2}B_{f}\end{bmatrix}e_{y}(t) +\begin{bmatrix}e(t) \\ e_{f}(t)\end{bmatrix}^{T} \begin{bmatrix}P_{1}C \\ P_{2}B_{f}G\end{bmatrix}v(t) +2\begin{bmatrix}e(t) \\ e_{f}(t)\end{bmatrix}^{T} \begin{bmatrix}P_{1} &{} 0 \\ 0 &{} P_{2}\end{bmatrix}^{T} \begin{bmatrix}e(t) \\ e_{f}(t)\end{bmatrix} \\&\quad \quad +[\tilde{H}\xi (t)-D_{f}Ff(e(t))-D_{f}G(t)]^{T}[\tilde{H}\xi (t)-D_{f}Ff(e(t))-D_{f}G(t)] -\gamma ^{2}_{0}v^{T}(t)v(t) \\&\quad \quad +(De(t)+Ff(e(t))+Gv(t)-e_{y}(t))^{T}\Psi (De(t)+Ff(e(t))+Gv(t)-e_{y}(t)) -e_{y}^{T}(t)\Phi e_{y}(t)\\&\quad \le \tilde{\eta }^{T}(t)(\tilde{\Omega }_{1}+\tilde{\Omega }_{2}^{T}\tilde{\Omega }_{2} +\tilde{\Omega }_{3}^{T}\Psi \tilde{\Omega }_{3})\tilde{\eta }(t) \end{aligned}$$

where $$\tilde{\eta }(t)=\begin{bmatrix} e(t) \\ e_{f}(t) \\ f(e(t)) \\ e_{y}(t) \\ v(t)\end{bmatrix}$$,  $$ {\tilde{\Omega }_{1}=\begin{bmatrix} P_{1}A+A^{T}P_{1}+2P_{1}+\iota _{1}L_{1}^{T}L_{1} +(\iota _{1}^{-1}+\iota _{2}^{-1})P_{1}MM^{T}P_{1} &{} D^{T}B_{F}^{T} &{} P_{1}B &{} 0 &{} P_{1}C\\ *&{} A_{F}+2P_{2} &{} B_{F}F &{} -B_{F}^{T} &{} B_{F}G \\ *&{} *&{} -\iota _{2}L_{2}^{T}L_{2} &{} 0 &{} 0 \\ *&{} *&{} *&{} -\Phi &{} 0 \\ *&{} *&{} *&{} *&{} -\gamma _{0}^{2}I \\ \end{bmatrix}}$$, 

$$\tilde{\Omega }_{2}=\begin{bmatrix} H-D_{F}D&-C_{F}&-D_{F}F&0&-D_{F}G \end{bmatrix}$$,  $$\tilde{\Omega }_{3}=\begin{bmatrix} D&0&F&-I&G \end{bmatrix}$$,  $$A_{F}=P_{2}A_{f}$$,  $$B_{F}=P_{2}B_{f}$$,  $$C_{F}=C_{f}$$,  $$D_{F}=D_{f}$$.

The last step in Eq. (18) also utilizes Lemma 2, which we would not repeat here. Then, using the Schur Complement Lemma three times for Eq. (15) and collapsing yields $\tilde{\Omega }_{1}+\tilde{\Omega }_{2}^{T}\tilde{\Omega }_{2} +\tilde{\Omega }_{3}^{T}\Psi \tilde{\Omega }_{3}<0$, and thus Eq. (16) holds. The proof is completed. $\square$

The following Theorem is considered to get a smaller performance index. It’s a suboptimal and better result in state estimation.

### Theorem 3

*For the system* (14) *if it can find positive scalars* $\iota _{1}$, $\iota _{2}$, *and a positive symmetric matrices* $P_{1i}\in \mathbb {R}^{n\times n}$, $P_{2j}\in \mathbb {R}^{n_{f}\times n_{f}}$, $\Phi \in \mathbb {R}^{m\times m}$, $\Psi \in \mathbb {R}^{m\times m}$ matrices $A_{F}\in \mathbb {R}^{n_{f}\times n_{f}}$, $B_{F}\in \mathbb {R}^{n_{f}\times m}$, $C_{F}\in \mathbb {R}^{p\times n_{f}}$, and $D_{F}\in \mathbb {R}^{p\times m}$ such that

$$\begin{aligned} \begin{aligned}{}&\min ~~ \gamma _{0} \\ s.t.&~~(15)~~holds, \end{aligned} \end{aligned}$$

*then it could be find a suboptimal* $H_{\infty }$ *performance index* $\gamma _{0}$.

In addition, to avoid triggering the sample an infinite number of times in a short period of time, i.e. Zeno behavior, the following theorem will give a positive lower bound on the event trigger interval.

### Theorem 4

*For Eq*. (12), *assume that exist positive scalars* $\bar{m}$, $\bar{n}$, $\bar{p}$, $\bar{q}$, *and* $\bar{r}$ *such that* $\frac{\Vert e(t)\Vert }{\Vert e(t_{k})\Vert }\le \bar{m}$,  $\frac{\Vert f(e(t))\Vert }{\Vert e(t_{k})\Vert }\le \bar{n}$,  $\frac{\Vert v(t)\Vert }{\Vert e(t_{k})\Vert }\le \bar{p}$,  $\frac{\Vert \dot{f}(e(t))\Vert }{\Vert x(t_{k})\Vert }\le \bar{q}$,  $\frac{\Vert \dot{v}(t)\Vert }{\Vert e(t_{k})\Vert }\le \bar{r}$,  *then the lower bounded of the minimum event-trigger inter-execution interval is*

$$\begin{aligned} T_{\min }=\min \{t_{k+1}-t_{k}\}>0, \end{aligned}$$

*where* $$t_{k+1}-t_{k} \ge \frac{\Vert \Psi \Vert \Vert D\Vert }{\Vert D\Vert [(\Vert A\Vert +\Vert ML_{1}\Vert )\bar{m}+(\Vert B\Vert +\Vert ML_{2}\Vert )\bar{n}+\Vert C\Vert \bar{p}] +\Vert F\Vert \bar{q}+\Vert G\Vert \bar{r}}$$.

### Proof

For any $0<t\in [t_{k}, t_{k+1}$, then $\Vert e_{y}(t)\Vert =\Vert y(t)-y(t_{k})\Vert <\Vert \Psi \Vert \Vert y(t_{k})\Vert$ from (12). For any $t\in [t_{k}, t_{k+1})$, it holds

$$\begin{aligned} \begin{aligned} \Vert \dot{e}_{y}(t)\Vert \le&\Vert \dot{y}(t)\Vert \\ =&\Vert D[(A+\Delta A(t))e(t)+(B+\Delta B(t))f(e(t))+Cv(t)]+F\dot{f}(e(t))+G\dot{v}(t)\Vert \\ \le&\Vert D\Vert [(\Vert A\Vert +\Vert ML_{1}\Vert )\Vert e(t)\Vert +(\Vert B\Vert +\Vert ML_{2}\Vert )\Vert f(e(t))\Vert +\Vert C\Vert \Vert v(t)\Vert ] +\Vert F\Vert \Vert \dot{f}(e(t))\Vert +\Vert G\Vert \Vert \dot{v}(t)\Vert \\ \le&\{\Vert D\Vert [(\Vert A\Vert +\Vert ML_{1}\Vert )\bar{m}+(\Vert B\Vert +\Vert ML_{2}\Vert )\bar{n}+\Vert C\Vert \bar{p}] +\Vert F\Vert \bar{q}+\Vert G\Vert \bar{r}\}\Vert e(t_{k})\Vert \\ \end{aligned} \end{aligned}$$

Taking the time integral from $t_{k}$ to *t*, one has

19 $$\begin{aligned} \begin{aligned} \Vert e_{y}(t)\Vert -\Vert e_{y}(t_{k})\Vert \le \{\Vert D\Vert [(\Vert A\Vert +\Vert ML_{1}\Vert )\bar{m}+(\Vert B\Vert +\Vert ML_{2}\Vert )\bar{n}+\Vert C\Vert \bar{p}] +\Vert F\Vert \bar{q}+\Vert G\Vert \bar{r}\}\Vert e(t_{k})\Vert (t-t_{k})\\ \end{aligned} \end{aligned}$$

When $t=t_{k+1}$, it triggers the homologous event, that signifies

$$\begin{aligned} \begin{aligned} \Vert e_{y}(t_{k+1})\Vert =&\Vert \Psi \Vert \Vert y(t_{k})\Vert = \Vert \Psi \Vert \Vert De(t_{k})+Ff(e(t_{k}))+Gv(t_{k})\Vert \ge \Vert \Psi \Vert \Vert D\Vert \Vert e(t_{k})\Vert . \end{aligned} \end{aligned}$$

And by $\Vert e_{y}(t_{k})\Vert =0$ and Eq. (19), it follows

$$\begin{aligned} \begin{aligned} \Vert \Psi \Vert \Vert D\Vert \Vert e(t_{k})\Vert \le&\{\Vert D\Vert [(\Vert A\Vert +\Vert ML_{1}\Vert )\bar{m}+(\Vert B\Vert +\Vert ML_{2}\Vert )\bar{n}+\Vert C\Vert \bar{p}] +\Vert F\Vert \bar{q}+\Vert G\Vert \bar{r}\}\Vert e(t_{k})\Vert (t-t_{k}).\\ \end{aligned} \end{aligned}$$

Suppose $\Vert x(t_{k})\Vert \ne 0$, then it yields that

$$\begin{aligned} t_{k+1}-t_{k} \ge \frac{\Vert \Psi \Vert \Vert D\Vert }{\Vert D\Vert [(\Vert A\Vert +\Vert ML_{1}\Vert )\bar{m}+(\Vert B\Vert +\Vert ML_{2}\Vert )\bar{n}+\Vert C\Vert \bar{p}] +\Vert F\Vert \bar{q}+\Vert G\Vert \bar{r}} >0. \end{aligned}$$

In summary, one can find a $T_{\min }=\min \{t_{k+1}-t_{k}\}>0$ that excludes Zeno behavior under the proposed event-triggered strategy (12). Proof is finished. $\square$

### Remark 1

Theorem 3 serves to rule out the possibility that the event-triggered strategy (Eq. 12) may have unlimited sampling for a short period of time, i.e., the Zeno phenomenon. If infinite sampling occurs in a short period of time, this will not only not reduce the number of samples, but also increase the burden of computer computation and measurement, or even computer crash, so the event-triggered strategy that excludes the occurrence of such a situation is good for in application of the considered event-triggered strategy.

## Simulation

**Figure 3:**
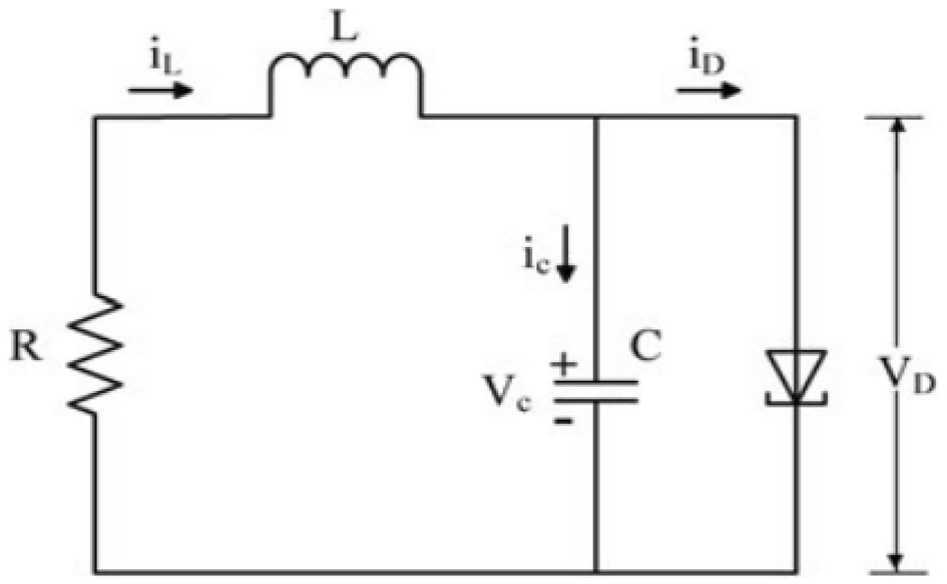
The tunnel diode circuit.

The well-known Tunnel Diode Circuit in Fig. 3 which modeled as the system (1) is illustrated to display the effectiveness of the method.

Assume the coefficient matrices are

$$\begin{aligned}{} & {} A=\left[ \begin{array}{lll} -5.1 &{} 1.5 \\ -7 &{} -2.1 \\ \end{array} \right] ,~ B=\left[ \begin{array}{lll} 1.3 &{} 1.2 \\ 1.0 &{} 0.2 \\ \end{array} \right] ,~ C=\left[ \begin{array}{l} 1 \\ 0.1 \\ \end{array} \right] ,~ D=\left[ \begin{array}{lll} 0.2 &{} 0.4 \\ \end{array} \right] ,~ F=\left[ \begin{array}{lll} 0.6 &{} 0.8 \\ \end{array} \right] ,~ G=0.4,~\\{} & {} H=\left[ \begin{array}{lll} 0.7 &{} 0.3 \\ \end{array} \right] ,~ M=\left[ \begin{array}{cccc} 10 &{} 10 \\ 0.2 &{} 1 \\ \end{array} \right] ,~ L_{1}=\left[ \begin{array}{cccc} -0.3 &{} -0.3 \\ 2.8 &{} 0.2 \\ \end{array} \right] ,~ L_{2}=\left[ \begin{array}{cccc} -0.1 &{} 0.1 \\ 2.2 &{} -0.5 \\ \end{array} \right] ,~ \\{} & {} N(t)=\left[ \begin{array}{cccc} \sin (t)\exp (-0.9t) &{} 0 \\ 0 &{} 0.75\exp (-t) \\ \end{array} \right] . \end{aligned}$$

Choose the neuron activation function as $$f(e(t))=\left[ \begin{array}{l} \tanh (e_{1}) \\ \tanh (e_{2}) \\ \end{array} \right] $$,  and $v(t)=-0.1\cos (t)\exp (-0.2t)$,  $\iota _{1}=1$, $\iota _{2}=0.8$, $\gamma _{0}=2$.

By Theorem 1, it can formulate the state estimator parameters as following:

1. The parameters of the full-order ($n_{f}=2$) $H_{\infty }$ state estimator for the system (1):
  $$\begin{aligned} A_{f}=\left[ \begin{array}{cccc} -2.9271 &{} -0.1689 \\ -0.4595 &{} -3.7192 \\ \end{array} \right] ,~ B_{f}=\left[ \begin{array}{cccc} -0.0003 \\ -0.0007 \\ \end{array} \right] ,~ C_{f}=\left[ \begin{array}{cccc} -1.0870 &{} -1.0870 \\ \end{array} \right] ,~ D_{f}=1.1930. \end{aligned}$$
2. The parameters of the reduced-order ($n_{f}=1$) $H_{\infty }$ state estimator for the system (1):

$A_{f}=-2.2704$,  $B_{f}=-0.0746$,  $C_{f}=-0.2562$,  $D_{f}=0.4232$. And by Theorem 2, it can formulate the state estimator parameters as following:

1. The parameters of the full-order ($n_{f}=2$) $H_{\infty }$ state estimator for the system (1):
  $$A_{f}=\left[ \begin{array}{cccc} -2.9335 &{} -0.1817 \\ -0.4888 &{} -3.7419 \\ \end{array} \right] $$,  $$B_{f}=\left[ \begin{array}{cccc} -0.0003 \\ -0.0007 \\ \end{array} \right] $$,  $$C_{f}=\left[ \begin{array}{cccc} 1.0495 &{} 0.3705 \\ \end{array} \right] $$,  $D_{f}=1.2059$,  and the event-triggered parameters are $\Phi =1.9985$ and $\Psi =0.0343$.

**Figure 4:**
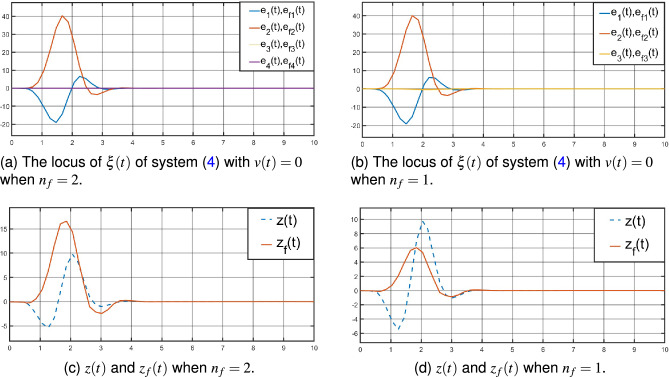
The dynamic trajectory by Theorem 1.
2. The parameters of the reduced-order ($n_{f}=1$) $H_{\infty }$ state estimator for the system (1):
  $A_{f}=-2.4452$,  $B_{f}=-1.5084$,  $C_{f}=-0.1620$,  $D_{f}=0.4845$,  and the event-triggered parameters are $\Phi =0.6467$ and $\Psi =0.2760$.

**Figure 5:**
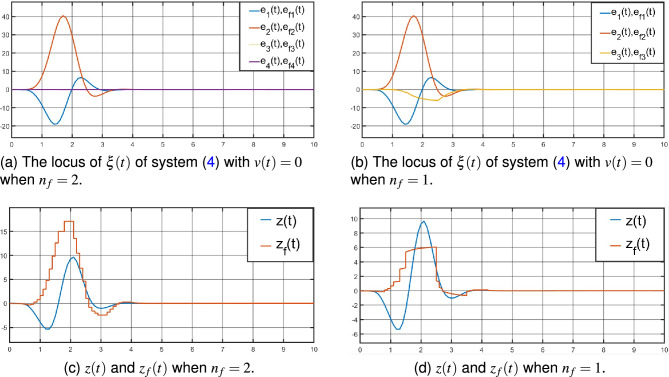
The dynamic trajectory by Theorem 2.

Then, by the SIMULINK Toolbox of MATLAB, the system (4) is exponentially stable with $v(t)=0$, see Fig. 4a, b by Theorem 1, Fig. 5a, b by Theorem 2. And from Fig. 4a, 5a for $n_{f}=2$, Figs. 4b, 5b for $n_{f}=1$, the locus of $\xi (t)$ of system (4) with $v(t)=0$ shows little difference in the overall trend. The oscilloscopes for *z*(*t*) and $z_{f}(t)$ is displayed in Fig. 4c, d by Theorem 1, Fig. 5c, d by Theorem 2.

From Figs. 4c, d, 5c, d above, compared with the full-order ($n_{f}=2$) and reduce-order ($n_{f}=1$) state estimators, the $z_{f}(t)$ of the reduce-order state estimator is closer to *z*(*t*), and the order is lower. This shows that a lower order state estimator can be designed under the premise of maintaining the same weighted $H_{\infty }$ performance index. And according to Figs. 4c, 5c, 4d and 5d, the state estimators given by Theorems 1 and 2 do not differ much in their estimation of *z*(*t*).

From Figs. 4c, d, 5c, d above, compared with the full-order ($n_{f}=2$) and reduce-order ($n_{f}=1$) state estimators, the $z_{f}(t)$ of the reduce-order state estimator is closer to *z*(*t*), and the order is lower. This shows that a lower order state estimator can be designed under the premise of maintaining the same weighted $H_{\infty }$ performance index. And according to Figs. 4c, 5c, 4d and 5d, the state estimators given by Theorems 1 and 2 do not differ much in their estimation of *z*(*t*).

**Figure 6:**
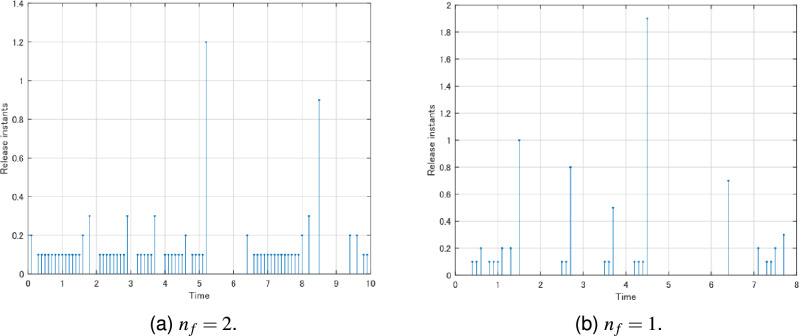
The evolution of the event-triggered sampled strategy (12).

However, as can be seen from Fig. 6a, b, the use of event-triggered sampling avoids the need to sample *y*(*t*) from time to time, which reduces the computational and time costs and saves resources. And compared with the existing studies, such as^1,2,4,5^, the reduce-order ($n_{f}=1$) state estimator and the event-triggered reduce-order ($n_{f}=1$) state estimator can reduce computational resources in by utilizing state estimators of smaller order instead of full-order state estimators to achieve the same goal in practical applications.

### Remark 2

The reduced-order filter can save the communication resources because the order of the filter state is reduced. In detail, the state dimension of the full-order filter is equal to that of the original neural networks, while the state dimension of the reduced-order filter is less than that of the original neural networks. In terms of the state dimension, the reduced-order filter saves communication resources. And in Fig. 6a, b, they are all event-triggered filter. The event-triggered reduce-order ($n_{f}=1$) state estimator can save communication resources because of the reduced order. Compared the event-triggered full-order filter ($n_{f}=21$) in Fig. 6a with the event-triggered reduce-order ($n_{f}=1$) state estimator in Fig. 6b, the reason of saving communication resources is same, i.e. the reduced order. However, for the filter without event-triggered strategy and the filter with event-triggered strategy, the filter that is not based on the event-triggered strategy receives is all *y*(*t*), while the filter based on the event-triggered strategy receives only the *y*(*t*) sifted by the event-triggered generator, thus realizing the saving of communication resources. Therefore, the reduce-order state estimator and the event-triggered reduce-order state estimator can reduce computational resources in by utilizing state estimators of smaller order instead of full-order state estimators. The method proposed in this paper has the following limitations: (1) In practical applications, it may be difficult to find reduced-order filters. (2) The eligible event-triggered strategy may miss the transmission of critical data, thus affecting the actual measurement and estimation. These are what we need to circumvent in our future research.

According to the above analysis, the proposed methods of the reduced-order $H_{\infty }$ state estimators and the (reduced-order) $H_{\infty }$ state estimators based on the event-triggered sampling strategy are availability.

## Conclusions

An $H_{\infty }$ state estimation has been studied for continuous-time neural networks with norm-bounded uncertainties via designing a reduced-order $H_{\infty }$ state estimator and an event-triggered reduced-order $H_{\infty }$ state estimator. For the considered neural networks with uncertainties, both a reduced-order $H_{\infty }$ state estimator and an event-triggered reduced-order $H_{\infty }$ state estimator have been designed, which ensure that the error dynamic is exponentially stable and has weighted $H_{\infty }$ performance index by Lyapunov function method. Moreover, it has also given the devised methods of the reduced-order and event-triggered reduced-order $H_{\infty }$ state estimator. compared with the full-order ($n_{f}=2$) and reduce-order ($n_{f}=1$) state estimators, the $z_{f}(t)$ of the reduce-order state estimator is closer to *z*(*t*), and the order is lower. The use of event-triggered sampling avoids the need to sample *y*(*t*) from time to time. Finally, by the example of well-known Tunnel Diode Circuit, it has shown that a lower order state estimator can be designed under the premise of maintaining the same weighted $H_{\infty }$ performance index, and using the event-triggered sampling strategy can reduce the computational and time costs in communication and save resources. And in the future, our direction is focus on the reduce-order ($n_{f}=1$) state estimators design for the neural networks with impulse and affine disturbance by applying an event-triggered strategy.

## Data Availability

The datasets used and/or analysed during the current study available from the corresponding author on reasonable request.
